# Locking Plate Fixation with Calcium Phosphate Bone Cement Augmentation for Elderly Proximal Humerus Fractures—A Single-Center Experience and Literature Review

**DOI:** 10.3390/jcm13175109

**Published:** 2024-08-28

**Authors:** Chun-Chi Peng, Ting-Han Tai, Chih-Yu Chen

**Affiliations:** 1 Department of Orthopedics, Taipei Medical University Shuang Ho Hospital, New Taipei City 23561, Taiwan; 23206@s.tmu.edu.tw (C.-C.P.); 19167@s.tmu.edu.tw (T.-H.T.); 2 Division of General Medicine, Department of Medical Education, Shuang Ho Hospital, Taipei Medical University, New Taipei City 23561, Taiwan; 3 International Ph.D. Program in Biomedical Engineering, College of Biomedical Engineering, Taipei Medical University, Taipei 11031, Taiwan

**Keywords:** elderly proximal humerus fracture, operative management, bone cement augmentation

## Abstract

Proximal humerus fractures (PHFs) are among the most common upper-extremity fractures, with a rising incidence linked to the growing elderly population. Treatment options include non-surgical and surgical methods, but the best approach for geriatric PHFs remains debated. Patient selection for treatment must consider clinical and functional outcomes and the potential complications of surgery. Osteoporosis, a key factor in elderly PHFs, meaning those in patients over 65 years old, often results from low-energy trauma and necessitates treatments that enhance bone healing. Bone cement, such as calcium phosphate, is widely used to improve fracture stability and healing. However, the benefits of surgical fixation with bone cement augmentation (BCA) for elderly PHF patients remain controversial. Hence, in this article, we searched databases including MEDLINE, Embase, the Cochrane Central Register of Controlled Trials, and Web of Science to analyze the evidence on locking plate fixation (LPF) with BCA for proximal humeral fractures. We aim to provide readers with updates concerning the above issues.

## 1. Introduction

Proximal humerus fractures (PHFs) are one of the most common types of upper-extremity fractures [1], accounting for about a third of upper-extremity fractures among the elderly population [2]. The prevalence of this kind of fracture increases with age and is more common in women, which could be linked to the higher incidence of osteoporosis in older women [3]. In fact, PHFs make up 18% of fractures in elderly patients with osteoporosis [4], and a 10% increase in PHF has been reported over the last decade [5]. However, whether geriatric patients should undergo surgical treatment still remains controversial. A multicenter randomized control trial compared operative and non-operative treatment for displaced two-part PHF; displacement was defined by Neer classification (displacement of more than 1 cm or 45 degrees, with bony contact) in patients 60 years of age or older, and no significant difference in clinical outcomes was found between the two groups [6]. On the other hand, Cosic et al. compared non-surgical and surgical treatment for PHF with 100% translation and demonstrated that operative management could result in better functional scores, fewer complications, and lower risk of non-union or malunion [7]. 

Various surgical treatments are available for treating PHF, including intramedullary nailing (IMN), percutaneous K-wire fixation, hemiarthroplasty (HA), and reverse shoulder arthroplasty (RSA), but the majority of patients still undergo locking plate fixation (LPF) [8]. Locking plate fixation has been reported to achieve satisfactory results even in elderly patients [9,10], but was unable to provide improvement in Disabilities of the Arm, Shoulder, and Hand (DASH) score [6]. Furthermore, a high complication rate was reported in several previous studies, ranging from 15.4 to 59% [10,11,12,13]. Therefore, to cope with common complications such as screw cutout or varus malreduction, the usage of bone cement augmentation might be a reasonable choice for reinforcing LPFs and thus yielding better clinical outcomes.

Over the past decade, locking plate fixation (LPF) for proximal humerus fractures (PHFs) with bone cement augmentation has shown significant clinical benefits, including improved mid- to long-term outcomes, higher union rates, and reduced complication rates compared to non-cemented techniques [14,15,16,17,18]. Various bone cements, such as PMMA (polymethyl methacrylate) and calcium phosphate, offer distinct advantages. PMMA provides strong stability but increases the risk of avascular necrosis around the screw tip [17,18,19,20]. In contrast, calcium phosphate cement avoids thermal injury, promotes osteoconductivity, and remodels gradually [21,22,23]. Overall, bone cement augmentation in LPF is particularly beneficial for elderly patients with osteoporosis, enhancing fracture stability and minimizing complications. Furthermore, biomechanical studies by Schöbel et al. and Kennedy et al. suggest that bone cement augmentation, particularly with calcium phosphate-based cements, enhances primary stability in locking plate fixation for proximal humeral fractures, potentially reducing intra-fragmentary motion and improving fixation strength [23,24,25].

In the present review, we reviewed the latest evidence in terms of current concepts of management and the use of locking plate fixation with bone cement augmentation for elderly PHFs. Databases including MEDLINE, Embase, the Cochrane Central Register of Controlled Trials, and Web of Science were searched for the related literature using different combinations of keywords including elderly, geriatric, proximal humerus fractures, complication, osteoporosis, bone cement, augmentation, and calcium phosphate. The two co-first authors then independently screened the titles and abstracts of potentially related literature and identified articles within the scope of this review. In addition, we share our clinical insights and suggested potential areas for future research related to geriatric PHFs. Our goal is for this article to offer readers a thorough and current overview of PHFs in the elderly population.

## 2. Management of Elderly PHFs

Treatment methods for PHFs can be divided into non-operative and operative treatments. However, there is still some controversy and discussion regarding which method should be used. Below is an analysis of these two treatment approaches.

### 2.1. Non-Operative Treatment 

Non-surgical treatments include a standard sling, shoulder spica cast, hanging arm cast, and airplane splint. These methods are significantly less expensive compared to surgical treatment. Recent studies, such as that of Lapner et al., indicate that surgical treatment with either locked plates or HA results in similar functional scores and pain outcomes as non-operative treatment [26]. Launonen et al. found no significant difference in clinical outcomes at 2 years between surgery and non-operative treatment in patients 60 years of age or older with displaced two-part fractures of the proximal humerus [6]. These studies suggest that non-surgical treatment for PHFs is as effective as surgical treatment and avoids post-surgical complications. However, non-surgical treatment also carries risks of non-union, malunion, and poor alignment [27].

### 2.2. Operative Treatment

According to the existing literature, operative treatment could provide a more effective reduction in pain [28] and may achieve a better coronal plane alignment at 3 months [29]. Likewise, Samborski et al. demonstrated that LPF led to decreased pain and improved passive ROM compared with non-operative treatment at 3 months [30]. Among several options of treatment for PHF, Klug et al. revealed that LPF was the most used procedure in 642,556 cases of PHF, accounting for 48.3% of all surgical treatment [8]. However, the suitable treatment of PHF is still debatable.

In a meta-analysis regarding surgical treatment of PHF, the clinical outcomes and range of motion in LPF were found to be superior to those in HA and comparable to those in IMN and RSA [31]. In treating two- to three-part PHF, both IMN and LPF led to high complication rates of 19% and 30%, respectively [32]. The main problem of IMN in three-part PHF is that the starting point is often compromised, so plate fixation may be feasible despite the high risk of screw cutout. With regard to more severe fractures, Launonen et al. compared non-operative LPF and HA for three- to four-part PHF in older patients (>60 years) and found no difference in DASH score, constant score, and OSS (Oxford shoulder score) upon between-group and within-group comparison [12]. Gadea et al. retrospectively compared IMN and LPF for four-part displaced PHF and found that when a medial hinge was present preoperatively, LPF should be recommended because it could lead to better functional outcomes [33]. In their study, the rate of complications including avascular necrosis (AVN) and screw penetration was comparable between IMN and LPF, but the revision rate was higher in the LPF (30%) than IMN (18.5%). 

Locking plate fixation is effective in achieving good reduction and union rates in PHFs. However, it carries several risks and complications. According to a meta-analysis of 3422 cases, complications occurred at the following rates: intra-articular screw penetration (9.5%), varus collapse (6.8%), subacromial impingement (5.0%), avascular necrosis (4.6%), adhesive capsulitis (4.0%), non-union (1.5%), and deep infection (1.4%) [34]. These complications tended to increase with the patient’s age and the complexity of the fracture [34,35,36,37,38]. Additionally, the presence of osteoporosis further exacerbates these risks. Studies have highlighted that older patients and those with more complex fractures are more susceptible to these adverse outcomes, emphasizing the need for careful consideration and management in such populations [39,40,41].

The indications for surgery in the treatment of PHFs and the type of surgery to be undertaken are still controversial and depend on the status of the patient and Neer’s classification. Although locking plate fixation is the most frequent modality of surgical intervention, studies have proven that LPF does not have lower rates of complications and variable functional outcomes under different circumstances. Hence, some research works have combined bone cement with LPF for maximum results to achieve skeletal stabilization and reduce the post-operative complications of bone cement. 

### 2.3. Outcome

After the treatment of proximal humerus fractures, various outcomes and functional tools are used to assess the success of treatment and the recovery of the patient. In this context, the constant score tool is more predominantly used with a frequency of 65%. This is then followed by the DASH score in 31% of cases and the American Shoulder and Elbow Surgeons (ASES) score in 18% of cases (see Table 1) [42].

The constant score is the most commonly used overall scoring system; it evaluates shoulder function through pain, activities of daily living, range of motion, and power. The DASH score, however, is a patient-reported questionnaire that measures the extent of disability and symptoms in upper limb disorders. It brings together physician-assessed and patient-reported components to measure shoulder function.

A number of studies have revealed different predictors influencing the prognosis of proximal humerus fracture. These include age, sex, socioeconomic status, and the extent of the fracture. Other prognostic factors include surgical factors and operator skill. Moreover, factors such as general health, comorbidities, and adherence to rehabilitation protocols are all associated with the recovery and functional prognosis. This knowledge helps to form a treatment plan for each individual and delineates realistic expectations from the recovery process [43,44,45,46,47].

**Table 1:** Outcome functional measure tools commonly used to evaluate the effectiveness of PHF treatment.

Outcome Functional Measure Tools	Content
Constant score [48,49]	Requires both patient and physician participation to assess subjective and objective measurements including pain (15 points), activities of daily living (20 points), strength (25 points), and the range of motion such as forward elevation, external rotation, abduction and internal rotation of the shoulder (40 points), with a total of 100 points. The higher the score, the higher the quality of the function.
Disabilities of the Arm, Shoulder, and Hand (DASH) score [50]	A patient-specific questionnaire containing 30 items including three main categories: (1) difficulty in performing physical activities due to issues in the shoulder, arm, or hand (21 items); (2) severity of symptoms like pain, tingling, weakness, and stiffness (5 items); (3) the impact of these problems on social activities, work, sleep, and mental state (4 items). Each item ranges from 1 (no difficulty in performing or no symptom) to 5 (unable to do or very severe symptom). The summed scores of the 30 items are converted to a 0-to-100 scale with the following formula: [(sum of score/n) − 1] × 25, where n is the number of completed responses, and a higher score indicates greater disability.
American Shoulder and Elbow Surgeons Shoulder (ASES) score [51]	The ASES score can be viewed as a 100-point scale that evaluates two dimensions of shoulder function: pain and performance in activities of daily living (ADL). The ADL categories range from 0 (unable to do) to 3 (not difficult at all).
Oxford shoulder score (OSS) [52]	Contains 12 items to be answered by the patient independently, focusing on pain and patients’ functionality in daily activities. There are five categories of response for every question, with a score from 1 to 5. The summed scores range from 12 (best) to 60 (worst).

## 3. LPF with Bone Cement Augmentation for Elderly PHFs

Recognizing that LPF alone often fails to achieve significantly better outcomes than conservative treatments, orthopedic surgeons are examining whether incorporating BCA can overcome the limitations associated with LPF. This section reviews the existing literature on the use of LPF with BCA for PHFs, especially in elderly patients. We also provide an overview of various types of bone cements. Our survey identifies several pertinent studies, including two meta-analyses [53] and three retrospective studies [54,55,56], whose main findings and appraisals are summarized herein.

### 3.1. Bone Cement Augmentation for Locking Plate Fixation

In the past decade, plate fixation with cement augmentation has been initiated and claimed to provide excellent clinical results in treating PHFs. The literature reports benefits from the use of different bone cements. Longo et al. conducted a meta-analysis comparing the outcomes of patients who underwent locking plate fixation with cement augmentation or bone graft augmentation versus those who underwent locking plate fixation without augmentation. The study included 19 studies; 120 patients received locking plate fixation with bone graft augmentation, 179 patients received locking plate fixation with cement augmentation, and 336 patients received locking plate fixation without augmentation. Their findings suggest that locking plate fixation with cement augmentation resulted in a lower complication rate compared to locking plate fixation alone [53]. Another meta-analysis, which included a total of 541 patients, showed that cement augmentation may reduce overall complications primarily by preventing implant-related complications [57]. Gavaskar et al. injected calcium phosphate into the metaphyseal bone void after reduction in elderly patients with complex humeral fractures, reporting a 92% union rate [54]. Hristrov et al. used PMMA-based cement in three- and four-part PHF with good clinical results at 12 months, even in aged patients [58]. She et al. reported that a dedicated locking plate with cement-augmented screws showed good mid- to long-term results without complications such as humeral head necrosis or loosening of screws [14]. Cement augmentation in second-generation locked plating by Schuetze et al. showed reduced risks for complications and secondary dislocation [15]. Hakimi et al. showed that the rate of complications including loss of reduction and screw cutout was significantly lower with cement augmentation [16]. Foruria et al. and Siebenburger et al. both showed that elderly patients have a substantially lower rate of implant failure and a less frequent rate of loss of fixation using cement augmentation [17,18].

Comparing different types of bone cement, PMMA is used more frequently for its superior stability, yielding a low rate of displacement and loss of fixation in elderly patients with poor bone quality [18,20]. However, it may increase the risk of avascular necrosis around the screw tip, with incidence ranging from 3.8% to 16.7% [17,18,20]. On the other hand, calcium phosphate bone cement (CPBC) prevents thermal injury, promotes osteoconductivity, and gradually remodels. It mimics the mineral phase of bone, with compressive strength greater than normal cancellous bone [21], and therefore reduces stress, improving the stability between the implant and proximal humerus. Experience-based studies by Gavaskar et al., Kennedy et al., and Schöbel et al. [22,23,54] also show equivalent or even better results for CPBC. This reabsorption of the CPBC within six months to 10 years minimizes potential damage due to intra-articular cement leakage [59]. In brief, bone cement augmentation in plate fixation for PHF enhances stability. It reduces complications, especially in elderly patients with osteoporosis, by using the inherent benefits of different bone cements.

There are three related retrospective studies articles discussing the comparison of PHFs with or without calcium phosphate bone cement augmentation (CPBCA) (see Table 2). Egol et al. reviewed 92 patients who received a dedicated locking plate for PHFs, with 27 of them using calcium phosphate cement for augmentation. In comparison to the allograft cancellous chip augmentation group (*n* = 29) and the non-augmented group (*n* = 36), CPBCA demonstrated a significantly lower screw penetration rate [56]. However, the author did not record the patient-reported outcome. Knapp et al. compared bone defects augmented with CPBCA with those with empty defects (EDs) in humerus, radius and tibia fractures, including 11 PHFs treated with osteosynthesis with CPBCA and 11 treated with osteosynthesis alone [55]. The results indicated fewer complications in the CPBCA group relative to the ED group, which was also the case in geriatric patients. Though they did not conduct a head-to-head comparison between the case with PHF, the study still demonstrated the benefits of accelerating osseous healing and lowering post-operative complications with the application of CPBCA.

**Table 2:** Studies investigating the effectiveness of LPF with or without BCA for PHFs.

Study	Study Type	Subject	Intervention	Comparator	Outcome Measurements	Main Results	Conclusion
Knapp et al. [55]	Retrospective cohort	22 proximal humerus fracture (mean age: 55.44 years)	osteosynthesis with CPBCA (*n* = 11)	osteosynthesis alone (*n* = 11)	Complications; Radiographics; Fracture edge; Fracture gap; Articular surface	CPBCA group significantly reduces post-operative complications, including pseudarthrosis, post-traumatic arthrosis, and neurological diseases, compared to non-augmented treatments.	CPBCA have proven their clinical integrity by demonstrating safety in medical applications. They support an accelerated early bone healing process and reduce the severity of complications within the group of patients treated with these substitutes.
Egol et al. [56]	Retrospective cohort	92 acute traumatic proximal fracture (mean age: 61 years)	LPF with CPBCA (*n* = 27)	Cancellous chips (*n* = 29); no augmentation (*n* = 36)	Complications; Intra-articular penetration; Humeral head settling	CPBCA in LPF reduces humeral head settling and joint penetration compared to non-augmented repairs or repairs with cancellous chips.	CPBCA in the treatment of PHFs with LPF decreased fracture settling and significantly decreased intra-articular screw penetration.
Gavaskar et al. [54]	Retrospective cohort	29 displaced 3- to 4-part complex humeral fracture (mean age: 79 years)	Subchondral metaphyseal bone voids were filled with injectable CPBCA (*n* = 29)	nil	Radiological outcomes; Functional outcomes; Complications	In a follow-up of 26 patients over an average of 27 months, fractures united in 24, while 3 required arthroplasty due to complications. Functional improvement was shown by age-adjusted Constant (63.1 ± 11.9) and ASES (62.58 ± 7.5) scores.	Osteosynthesis with second generation locked plating techniques provide satisfactory outcomes in very elderly patients with complex PHFs with minimal complications.

LPF: locking plate fixation, BCA: bone cement augmentation, PHFs: proximal humeral factures, CPBCA: calcium phosphate bone cement augmentation.

### 3.2. Our Experiences

In our hospital, we retrospectively analyzed all the elderly patients over 65 years who had presented with a proximal humeral fracture due to low-energy injury. These patients were subsequently treated using the Biomet A.L.P.S. Proximal Humeral Plating System^®^ (Zimmer Biomet, Warsaw, IN, USA) reinforced with CPBCA (Pro-Dense^®^, Wright Medical Technology™, Arlington, TN, USA) between May 2023 and December 2023. Patients with debilitating pre-existing comorbidities, ipsilateral upper extremity injury, previous shoulder injury, delay in surgery of >two weeks, or who received <1 mL injected CPBCA were not included in this analysis. These exclusion criteria were used for the sake of homogeneity and result reliability.

With the application of these criteria, eight patients under 65 years of age and three patients who presented with only surgical neck fractures were excluded. This led to a group of 20 patients for the analysis: 6 men with an average age of 74.5 years (age range: from 66 to 88) and 14 women with an average age of 86.6 years (age range: from 82 to 94). No intraoperative complications occurred during surgery, showing a high level of safety in the procedure. Immediate post-surgical radiographs revealed 16 patients to have anatomic reduction, whereas the rest had near-anatomic reduction. This implies that surgical interventions managed to restore the anatomical alignment of the fractured humerus.

In no case was fixation failure, screw cut-through, or loss of fixation observed at both the 3-month and 6-month post-surgical follow-ups. This represents a valuable finding in establishing the stability and effectiveness of the combined treatment approach. The mean VAS scores, being explanatory measures of pain levels, were very low and amounted to 1.66 with a range of 0 to 6 at 3 months and 0.42 with a range of 0 to 4 at 6 months. These low scores reflect success in the pain management and recovery of the patient. No patient reported decreased shoulder motion or functional interference in daily life, which would suggest that no patient suffered from limited shoulder mobility. Three patients, however, developed minor secondary plate/screw displacement due to a fracture site compression. Even so, none of these needed surgical revision (see Table 3).

No post-operative complications, such as non-union or surgical site infection, were seen in the follow-up period. This confirms the safety and effectiveness of the treatment protocol followed. The follow-up radiological assessment showed great improvement in the diameter of the humeral head, position of the greater tuberosity, and the neck–shaft angle for each patient when compared to the first post-surgical radiograph. These changes are indicative of anatomical and functional recovery in the patients.

**Figure 1:**
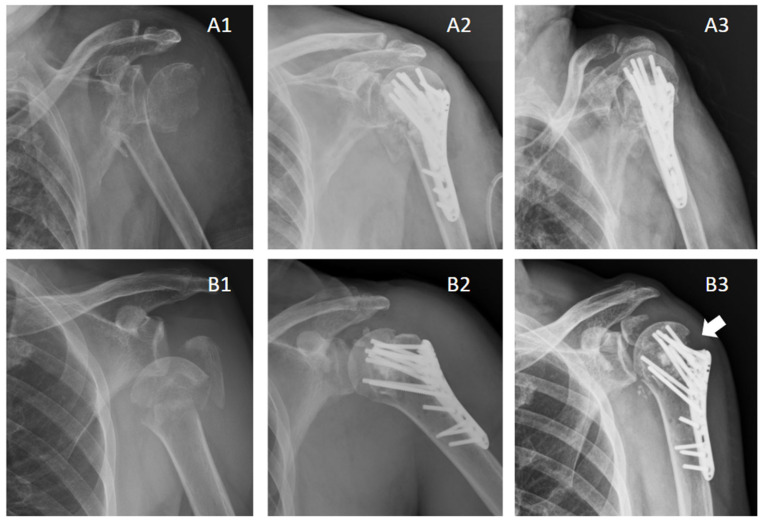
Comparison of LPF with (**A1**–**A3**) or without (**B1**–**B3**) CPBCA in elderly PHFs: Patient A: (**A1**) A left PHF was presented. (**A2**) Treated with LPF with CPBCA. (**A3**) Post-operative X-ray at 12 months follow-up; Patient B: (**B1**) A left PHF was presented. (**B2**) Treated with LPF without CPBCA. (**B3**) Post-operative X-ray at 12 months follow-up. By comparison, the patient managed with a combination of LPF and CPBCA fared better with a stable head of humerus, maintained neck–shaft angle, and position of greater tuberosity fragments; in patient B, who had LPF without CPBCA, the head of humerus had a progressive collapse, the neck–shaft angle was reduced, and greater tuberosity fragments were migrated (arrow).

The seniors have more prevalence of osteoporosis and are vulnerable to comminuted PHFs; traditional LPF alone cannot stabilize the post-operative radial height and inclination in many patients, especially when severe osteoporosis and serious metaphyseal bone loss is considered. However, through experience, the CPBCA is much more biomechanically supportive and eradicates the chances of secondary collapse with promotion of bone healing. Moreover, it can be appreciated in Figure 1 that the configuration is better retained with less chance of loss of fixation, delayed union, or non-union in the application of CPBCA in the LPF. This combination treatment seems to have special benefits for the restoration of structure and function in elderly patients with PHF.

## 4. Summary

In conclusion, based on this review, conservative non-surgical treatments have clinical and functional outcomes similar to those of surgical treatments with complication rates for elderly patients with proximal humerus fractures. However, the surgical treatments, including LPF, can achieve better anatomical reconstruction, reduce pain effectively, and improve passive range of motion compared with non-operative approaches in these appropriately selected elderly and super-elderly patients. Although LPF is one of the most common methods for the treatment of PHFs, the best surgical approach varies with individual patient characteristics.

With regard to post-operative outcomes, several complications are associated with LPF, such as intra-articular screw penetration, varus collapse, subacromial impingement, avascular necrosis, adhesive capsulitis, non-union, and deep infection. The major cause for all these complications is osteoporosis. LPF with BCA is much more beneficial for the elderly with osteoporosis as it increases the stability of the fracture and decreases complications. Clinical outcomes indicate that patients treated with this combination experience quicker recovery times and improved range of motion in the affected limb. The addition of CPBCA adds stability and enables early mobilization, thereby considerably shortening the overall rehabilitation period. The method also reduces the risk of post-operative complications, thus increasing the quality of life of patients during the phase of recovery. The evidence suggests that this innovative approach could set a new standard in the treatment of complex fractures in osteoporotic patients. Therefore, we do recommend detailed assessment of the individual condition of all elderly patients before initiating LPF with BCA.

However, since there is variation in most studies as to the type of bone cement used, additional RCTs with a larger sample size are necessary to confirm this beneficial effect of LPF with BCA in elderly PHFs. A subgroup analysis should also be conducted to tease out populations that specifically benefit more from LPF with BCA with respect to factors such as age, gender, activity levels, comorbidities like osteoporosis, and Neer classification. Further studies need to be conducted on the comparative effectiveness of surgical compared with conservative treatment in elderly patients with PHFs in terms of risks and outcomes in a more comprehensive way, integrating the relevant risk and predictive factors of optimizing resource allocations in keeping with patient preferences and expectations.

A few limitations of our review need to be mentioned. First, although we tried to write an up-to-date review on this topic, some of the studies may already have been published after the search time concluded. Second, due to the paucity of and heterogeneous research designs in studies, we cannot currently make a firm recommendation on LPF with BCA for elderly PHFs. Nevertheless, based on our clinical experiences, LPF with BCA seems effective and safe.

Therefore, LPF with BCA might provide better short-term and long-term results than non-surgical management for elderly patients with PHFs. More research in this area will be indispensable in determining the precise efficacy of LPF with BCA, developing improved assessment tools, and validating procedural approaches to treating elderly patients with PHFs.

## Figures and Tables

**Table 3:** Elderly patients with PHFs assessed between May 2023 and December 2023.

	Overall
No. of patients (male/female)	20 (6/14)
Mean age at surgery (range), y	74.5 (66–88)/86.6 (82–94)
VAS score (3 months post operation)	1.66 (0 to 6)
VAS score (6 months post operation)	0.42 (0 to 4)
Fixation failure	0
Decreased range of motion	0
Secondary plate/screw displacement	3

## Data Availability

Not applicable.
